# Clinical Lecture on Aphonia and Stammering

**Published:** 1849-01

**Authors:** Charles A. Lee


### ARTICLE V.

*Clinical Lecture on Aphonia and Stammering.* By Charles A. Lee, M. D. Delivered before the Medical Class of Geneva College.

Gentlemen:—The case presented before you is, in some respects, a remarkable one. This boy, who is now between four and five years of age, was attacked about a year since with fever, attended with a good deal of cerebral disturbance, manifested by delirium, &c., which persisted for several days. He soon, however, began to convalesce, and in a few weeks had recovered his usual degree of health. It was soon apparent, that he had entirely lost the faculty of speech. While convalescing, he was able to utter a few words, but he soon lost the power of articulation, and at the present moment, he is unable to utter a single intelligible sound; although, previous to his sickness, he was as talkative as boys generally are at his age. The parents have sent him here, from a distance, to see whether something can not be done for him, and it is our duty to examine the case, therefore, as thoroughly as possible, to ascertain if the defect may be remedied. In the first place, I would observe that the hearing of our little patient is as acute as it ever was, he understands apparently what is said to him; does as he is ordered; and judging from the expression of his features, we should pronounce him possessed of some considerable degree of intelligence.

You are aware that the formation of perfect vocal tones presupposes the possession of the sense of *hearing;* persons are dumb *because* they are deaf. They may utter a series of harsh sounds, (I speak of those born deaf) but even this is accomplished with the greatest difficulty; they can, to a very imperfect degree, learn the movements of articulation by means of their sight, so that it is not uncommon, to find some deaf persons, who understand what is said, by watching the motions of the lips and tongue of the speaker, but yet, if born deaf, such individuals, can rarely utter more than some harsh sounds like the inferior animals, for the want of hearing to regulate their articulation. For this reason, most of the attempts to teach the deaf and dumb to speak, have hitherto proved unsuccessful, although some instances are recorded, in which deaf persons have been able to carry on conversation in the regular way, regulating their voices by their voluntary power, guided by muscular sensation. It is not, then, from deficiency, or absence of this faculty, that our patient is unable to articulate. Vocal sounds, you are aware, are formed in the larynx; whilst their modifications by which language is formed, are effected in the oral cavity. Now, if there be, from any cause whatever, an inability of producing sound, you will necessarily have an absence of speech, which is sound articulated. In cases of ulceration, or relaxation of the vocal cords—those ligaments of the laryinx, by means of which sound is generated; or, if there be no defect in the power of producing sound, if you have malformation of the oral cavity, or paralysis of the ninth nerve, you will still have absence of speech.

But in the present case there is no abnormal conformation of the organs of speech; there is no organic change in the laryngeal structure, for he is capable of uttering loud sounds, as well as a variety of sounds—there is, apparently, no paralysis of the ninth nerve, on which the movements of speech, principally, depend, supplying, as it does, all the nerves of the tongue; there is no want of nervous supply in the lips, the movements of which are essential to distinct articulation, and which are supplied by the facial, a branch of the fifth nerve. To what cause, then, must we attribute the misfortune of our patient?

There is something else wanting besides what I have stated, in order that we have the faculty of speech. It is *mind*—speech is but the expression of ideas; ideas are the conceptions of an intelligent being; he may, from disease, have los the faculty of reason and understanding; still he has the power of speech; often incoherent—often unintelligible—often a strange jargon of unmeaning words,—still it is speech. Now, there may be temporary or permanent want of speech from cerebral defect or derangement, from malformation, or organic lesions in that wonderful organ which is essential to the existence of ideas, of which words are the outward manifestations.

A man struck down by an apoplectic attack, does not speak, for the physical condition of his brain will not allow it to work; it is a state incompatible with its normal functions; the will, and all the mental faculties are suspended, and consequently, the action of all the muscles dependent on the will; those of the voice equally with the rest. So, also, in the stupor from concussion, the coma from narcotics, and poisonous gases. But this is not a case of this kind. Speech, then, is an intellectual process. The infant has the same vocal organs as the adult, but it is only when the intellect has been sufficiently developed to enable him to understand the meaning of the sounds that he hears, that he undertakes to utter similar sounds, and so, by imitation, he acquires the faculty of speech. The voice of the idiot may be strong and his hearing acute, but he is incapable of speech. In short, we must seek for the organ of the faculty of language in the brain, and regard the ear, the larynx and the vocal tube as its instruments. The ourang-outang has the vocal organs of man, but it has not his intellectual faculty of language. You see the boy is active, if not sprightly, but he is mute. He seems pleased with the merest trifles, he smiles continually, without any apparent cause; is it probable that he is idiotic? Such is my conviction. Enquiry satisfies me that his intellect is gone, not that his mind is an entire blank. But his is a condition bordering on that of some of the inferior animals, which, though possessing a certain amount of intelligence, and some of the faculties which we are too apt to suppose belong solely to man, are yet incapable of expressing their wants, or their instincts, by articulate speech; such, I say, is my conviction, after all the light I have been able to gather both from personal examination and facts communicated by his friends. Of course, our art is valueless in such a case as this. Its resources are inadequate to reach the results of organic changes, like these; all we can do is to hope that a gradual amelioration and improvement may be affected by the lapse of time, and that the vis medicatrix, may, in her silent, constant, and mysterious workings, repair the deranged cerebral organization, and build up healthy in the place of diseased structure. As his general health is perfect, I shall make no prescription, except to advise kindly treatment, and attention to those hygienic influences so essential to corporal and mental well being. Let his food be nourishing and of easy digestion; let him breathe a pure air by day and by night; let his clothing be properly regulated; his skin kept in a healthy state by frequent ablutions of cold or tepid water, and dry friction; let every effort be made to draw out, develope, and strengthen the mental faculties, for, on the success of this, hangs the entire result. The term aphonia is strictly applicable only to those cases of suppression of speech, which takes place independently of coma or syncope. It may be consequent on the tumefaction of the fauces and glottis, tumors connected with the trachea, ulcerations and relaxation of the *chordæ vocales*, as well as mechanical division or paralysis of the nerves distributed to the tongue and larynx. In all these cases, loss of voice is purely a sympathetic affection, and, probably, it is always so, for we can scarcely conceive of its being idiopathic. Many persons, if attacked with catarrh, are liable to lose their voice for a time, owing to a thickening of the lining membrane of the larynx, and the same accident is a common occurrence in chronic bronchitis. It is a very common symptom, also, in chronic laryngitis, and laryngeal phthisis, especially in its latter stages. Aphonia is very frequently dependent on atony or relaxation of the vocal chords, from over exertion in speaking, singing, or shouting, sometimes connected with particular disease of the mucous membrane, while in others, this complication does not exist. Loss of voice is not an unusual occurrence, also, in cases of syphiltic ulcerations, which attack the cartilages and soft parts of the larynx, making great havoc, and such cases are usually attended with rapid emaciation, profuse expectoration of frothy mucus, hectic fever, and the other phenomena which commonly attend laryngeal phthisis. I have alluded to its occurrence in hysteria, owing to irregular distributions of the nervous power; there we have no structural lesion, as in the former instances, no palpable disorder of the organs of speech; but it grows out of a state of general irritability, which is only to be cured by tonics and strengthening regimen. I have already alluded to the aphonia which precedes or succeeds apoplexy, indicative of cerebral pressure or loss of nervous power—always a grave accident, and not to be disregarded with impunity.

The treatment of this affection, like that of every other, must always be founded on its pathology. Its causes must be diligently sought, and, if possible, removed; when it originates from catarrh, you will find an emetic of oxymel of squills with ipecacuanha often successful, following its use by external revulsives and internal demulcent and expectorant medicines. When it arises from an acute inflammatory condition of the lining membrane of the larynx, you will often succeed in restoring the voice by free leeching to the throat, and the use of moderately stimulating gargles.

A solution of the nitrate of silver, of the strength of 40 grains to the ounce, has perhaps more uniformly succeeded in curing aphonia, when caused by inflammation, relaxation, or ulceration of the mucous membrane of the larynx, than any other single remedy. It is applied by means of sponge, attached to a piece of whalebone, bent about an inch from the end to an angle of some 120 degrees; this is passed freely down to the vocal ligaments, after depressing the tongue by a curved spatula, and if the patient is directed to inflate the lungs fully, before passing the instrument, there will be no danger of any accidents, such as spasms, &c. You will find many cases treated successfully after this manner, by Drs. Green, Taylor and others, recorded in the pages of the New York Journal of Medicine. You will there find a case recorded of aphonia of two years standing, with all the signs of ulceration of the vocal ligaments, cured in a short time by the daily application to the larynx, of a solution of nitrate of silver of from 40 to 60 grs. to the ounce of water, the cough being, at the same time greatly relieved. In another case of chronic laryngeal affection, with total loss of voice for two months, the affection was cured by six applications. It is not always, or perhaps generally necessary in these cases, to carry your sponge into the larynx, merely brushing the parts about the fauces will often answer equally well. This may be done with a large camel's hair brush dipped in the solution. The effect, like that of gargles, extends by contiguous sympathy down the larynx, producing contraction of the vocal chords, and an intonation of the voice. I believe in nearly all of these cases of temporary aphonia, there is a relaxation of these ligaments, and that any measure calculated to contract them, and thus render them more tense, will remove the difficulty. It certainly is so in colds, catarrhs, and chronic laryngitis, in which, however, there is often ulceration present, and I have no doubt the same conditions exist in the aphonia of delicate females, in whom all the tissues are lax and flabby. How often do we find the voice sink an octave or more from the effects of a cold; your knowledge of music and the tones produced by stringed instruments, must convince you, that under such circumstances, the pathology I have suggested, must be the true one. The effects of remedies also justify the same conclusions. How do stimulating gargles of cayenne, vinegar, &c., restore the voice, unless it is by giving tone to, and causing contraction of, these vocal strings? Nitrate of silver, galvanic electricity, and other stimulants, operate in a similar manner, and if leeches do good, it is in those inflammatory cases, where the mucous membrane is gorged with blood, and where tonicity can only be restored by emptying the capillaries, and bringing the organ back to its healthy and normal condition. I have often seen attempts made to cure this affection in delicate hysterical females, by local antiphlogistic measures, and external revullents, but always without success, while a course of general tonics, as iron, cold showering, and quinine, with the local application of the nitrate of silver, or stimulating inhalations, as the vapor of benzoin, aromatic vinegar, fumes of rosin, &c., have generally been attended with success. You will recollect that aphonia may be owing to simple debility of the laryngeal muscles, brought on by over exertion of the vocal organs; in such cases, rest will restore the voice, if attention be paid to the general health, without resorting to the use of local remedies. You will also bear in mind that the voice may suddenly be lost through the influence of any strong mental emotion, as joy, anger, fright. If you meet with such cases, especially if they occur in hysterical subjects, you need not be alarmed; the voice will return after the system has had time to rally, and recover from the shock it has sustained. Occasionally I have met with a case brought on by a sudden change from warm to a cold air; these require scarcely any interference, as an exemption may be purchased by a removal to a dry warm atmosphere. Remember, too, that aphonia may arise from intestinal irritation, especially in children, who sometimes suffer an attack from the irritation of worms; here, anthelmintics followed by tonics, will be the proper remedies. The difficulty is greater where it is owing to an affection of the par vagum, or the recurrent nerve; and irremediable when caused by an aneurismal or other tumor pressing on these nerves. The cause here being persistent, the disease will also be persistent. When the cause is of a temporary nature, the affection, for the most part, will also be temporary, subsiding spontaneously, soon after the cause has been removed. I have but a single remark more on this branch of our subject to offer, and that is, as aphonia frequently occurs as a symptom in acute diseases, it is only to be obviated by the abatement of the original malady. In such cases, it is always a grave sign, and very often a fatal one; indicating, as it certainly does, a profound cerebral affection. A restoration of speech is always hailed as a favorable token in acute complaints, and as a harbinger of convalescence.

In conclusion, allow me to call your attention to the importance of general treatment in local affections of this nature. If the general health be robust, individuals will rarely suffer from such attacks, and if they do, the local difficulty is easily removed. Far different, however, is it with those who are of delicate or broken constitutions, especially if they are obliged to exert the organs of voice in public speaking or singing; sooner or later they are sure to suffer an attack, and when once experienced, a relapse will be sure to follow, if the exciting cause continue to operate, unless the general health be reinvigorated.

In several chronic cases, I have succeeded in restoring the voice, by advising the patient to take the tour of Europe, or a long sea-voyage, or changing his occupation to one requiring less use of the vocal organs, as farming, &c. The free use of cold water by spunging the body, especially about the throat, followed by dry friction, has been attended with the best results. Freedom from mental anxiety is almost essential to a cure, for the parts about the glottis seem to be the centre of fluxion, and nervous irradiations, in all affections of the human system, as hydrophobia, hysteria, thymic asthma, &c. In short, the patient must, if possible, be free from mental inquietude; breathe a pure atmosphere; take abundance of exercise in the open air; live upon plain, nourishing, and easily digested food; avoid all alcoholic stimulants, as well as tea, coffee, and tobacco; I have no fear but that the disease if present, will speedily be removed, and that when once removed, if the same mode of life be continued, there will be very little if any danger of its return.

Here is a case of a different kind. There is not a total inability, but a difficulty of speech, which is indistinct and imperfect, because the sounds do not follow each other as they ought to do; it is a case of *stammering*—now this is a singular vocal phenomenon, one which has led to some curious speculations on the part of certain physiologists, and still more singular practice on the part of certain surgeons. While some have located the difficulty in the larynx, others have found it in the mouth, and so have set about to remedy it by cutting the genio-hyo-glossi muscles, whose office it is to pull the tongue downwards and backwards, and draw the hyoid bone upwards. How such an operation is to remedy the difficulty is not so obvious: it would seem to offer about as much chance of success as severing the *tendo-achilles* to cure the staggering of a drunken man.

Stammering, you know consists in a momentary inability to pronounce a consonant or vowel, or connect it with the preceding sounds. The difficulty may exist in any part of a word, the beginning or the middle; if the obstacle occur at the middle, then the first part is repeated several times before the latter half can be pronounced. This repetition of the commencement of a word is only a new attempt to overcome the obstacle, and the impediment will be the greater if the word begins with one of the explosive consonants, (b, d, q, p, t, k,) which do not admit of a continuous pronunciation until the formation of a vowel is attained, as in the word bitter, which is pronounced b-b-b-b-bitter. The continuous consonants are got along with much easier, such as m, v, n, g, r, l, or s. The difficulty would seem, sometimes, to consist in connecting the consonant with the succeeding vowel, sometimes in uttering the first consonant of a word, and if you closely watch a stammering person when attempting to speak, you will see that the difficulty seems to consist in the momentary closure of the glottis; the air, necessary for the articulation of the sound, does not escape from the larynx. The obstacle does not exist in the mouth; the individual has sufficient control of his lips and tongue, but he cannot command a sufficient quantity of air to produce the necessary sounds. This is undoubtedly owing to a spasmodic state of the muscles of the glottis, the *thyro-artenoid*, which bring the ligaments of the glottis into contact with each other, and this depends on a morbid association of the muscles of the larynx with the movements of the organs of articulation. The stammerer finds no difficulty at all in bringing his lips into position for uttering any of the consonants, but as he has no control over the glottis, he cannot regulate the passage of the air, by which the desired sounds are to be made, it stops at the larynx.

Just watch a stammerer while he attempts to speak. What frightful contortions! There is evidently a desperate struggle going on in the glottis; all the muscles of respiration are called into exercise to overcome the spasm which prevents expiration; the veins of the neck become distended, and the face flushed from congestion of the brain, while its muscles are violently distorted, owing to the same spasmodic effort. The cause of the phenomenon, then, is understood.

*Is there any cure for stammering?* I *believe* there is, but not in the Surgical Armamentaria, nor in the materia medica proper. We read that Demosthenes cured himself of stammering by speaking with a pebble under his tongue. Mrs. Leigh, who has had great success in curing this difficulty, has availed herself of this hint, and directs her patients to elevate the tongue, raising the point towards the palate. Stammerers will tell you, that if they allow the tongue to lie low in the mouth, they find it much more difficult to articulate than if it is somewhat elevated. It is an excellent plan in the treatment of these cases, to direct the patient to sing his words, and you know that persons who stammer can sing much better than they can talk. In this way the attention is directed more to the larynx, and its muscles are brought at length under such a degree of control, that the habit of stammering is nearly or quite overcome. If we could devise a method by which the glottis could be kept permanently open, I doubt not that the habit could be easily cured. I know no better way of voluntarily keeping it open than that recommended by Dr. Arnott, viz.: that the patient should connect all his words by an intonation of the voice continued between the different words. A still later mode of curing the difficulty, is that suggested by Muller viz: of reading sentences, in which all letters, which cannot be pronounced with a vocal sound viz. b, d, q, p, t, and k, are omitted, and only those consonants included, which are susceptible of an accompanying intonation of the voice; which should also be prolonged as in singing. This plan while it keeps the glottis open, combines articulation with vocalisation. After practising in this manner for a while the stammerer should then proceed to the mute and continuous consonant h, and the explosive sounds q, d, b, k, t, p. This mode of treatment, followed up, I believe will cure most cases of stammering, however bad they may be, but then it will require great perseverance on the part of the patient, and patience on the part of the instructor, if there be one; although I see no necessity for a teacher, after the principle has been fully explaned, and understood. The patient is to study very carefully the manner of articulating the different letters, and then pronounce them repeatedly, slowly, and analytically. As soon as he can master sentences from which the explosive consonants have been omitted, he is to pass on to others in which they are sparingly introduced, and so on to ordinary language. Confidence in himself, and in his ability to command the muscles of articulation is of the highest importance to the stammerer, and this can only be acquired in the manner pointed out, viz: overcoming obstacles by degrees, and proceeding step by step from that which is easy and practicable, to that which is more difficult.

Nov. 1848.

*Appendix to the above by Prof. Lee.*

A very singular case of loss of speech, somewhat resembling the above, has recently been presented at our Clinique. M. C., a little girl, three years of age, was bitten in the cheek when twenty months old, by a rabid cat. The wound healed speedily, but about five weeks from the occurrence of the accident, violent spasms came on, affecting nearly all the muscles of the body, and which continued at longer or shorter intervals from Sunday until Friday evening following. The spasms then ceased, and she gradually got better; but it was soon ascertained that she had lost the power of speech, for she had begun to talk considerably, and also the use of her limbs. The parents state that during the hydrophobic attack, she was several times so far reduced as to be supposed to be dead, and was actually laid out once or twice, when she recovered. She now makes a few harsh sounds, like the deaf and dumb, but cannot articulate, Her hearing is quick; her face is not wanting in intelligence; but she is remarkably susceptible to bright lights, and sounds; constantly in active motion, and little inclined to sleep, it often requiring two or three hours to calm her to rest. She has a constant propensity to stand on her head, and turn somersets, and she is employed a great deal of the time in such feats, traversing the room in a succession of somersets, with astonishing rapidity. If left alone for a few minutes, she is often found standing on her head in the rocking chair, her hands resting on the arms of the chair, while her feet are thrown up against the back of the same. The top of the head is preternaturally hot over a space of two or three square inches; her pupils are much of the time considerably dilated. Her appetite is good, as is also her general health.

The only rational explanation of the above case, appears to me to be that which attributes the loss of speech, and the other symptoms, to cerebral derangement, consequent upon the disease which caused the spasms, &c. Some organic change has occurred, incompatible with the normal exercise of the intellectual faculties, and reducing her to a state bordering on idiocy. The case is therefore, probably, a hopeless one; though it is impossible to predict, with certainty, what will be the result. Medical treatment, however, promises but little.—*Buff. Med. Jour.*

